# Choriocapillaris Dysfunction in Retinal Diseases: Multimodal Imaging Biomarkers, Pathophysiological Insights, and Emerging Clinical Applications

**DOI:** 10.3390/biomedicines14102173

**Published:** 2026-09-25

**Authors:** Feliciana Menna, Alessandro Meduri, Laura De Luca, Antonio Baldascino, Stefano Lupo, Enzo Maria Vingolo

**Affiliations:** 1 Department of Medical-Surgical Sciences and Biotechnologies, U.O.C. Ophthalmology, Sapienza University of Rome, Via Firenze 1, 04019 Terracina, Italy; 2 Department of Biomedical and Dental Science and of Morphological and Functional Images, University of Messina, 98122 Messina, Italy; 3 Ophthalmology Unit, Fondazione Policlinico Universitario A. Gemelli IRCCS, 00168 Rome, Italy

**Keywords:** choriocapillaris, optical coherence tomography angiography, flow deficits, choroid, age-related macular degeneration, geographic atrophy, pachychoroid, central serous chorioretinopathy, diabetic choroidopathy, imaging biomarkers

## Abstract

The choriocapillaris is the terminal capillary bed that sustains the retinal pigment epithelium (RPE) and the outer retina, and its integrity is inseparable from that of the RPE–Bruch’s membrane complex it supplies. Histopathological work established decades ago that choriocapillaris density declines with age and falls further in disease, but for most of that period, the layer could not be assessed reliably in living eyes. Optical coherence tomography angiography (OCTA), particularly with swept-source instrumentation, signal compensation and validated thresholding, has changed this: choriocapillaris flow deficits can now be quantified reproducibly and followed over time. A flow deficit is, however, a signal-level measurement that does not by itself establish anatomical capillary loss, and this distinction is maintained throughout. This review examines what that capability has revealed. We first summarize choriocapillaris anatomy, its functional coupling to the RPE through polarized vascular endothelial growth factor secretion, and the mechanisms implicated in its failure, including complement-mediated endothelial injury, Bruch’s membrane thickening, oxidative stress and venous overload. We then address the methodology in detail—slab definition, shadow compensation, binarization, averaging and the artefacts that inflate or mask flow deficits—because the comparability of published values depends almost entirely on these choices. The clinical sections review choriocapillaris involvement in age-related macular degeneration, where baseline flow impairment has been associated with subsequent drusen formation and with the rate of geographic atrophy enlargement, an association that is temporal rather than proven causal and that rests on a small number of cohorts; in the pachychoroid spectrum, where the layer is thought to be compressed rather than primarily diseased; and in diabetic retinopathy, myopia, inherited retinal dystrophies, inflammatory chorioretinopathies and systemic vascular disease. We conclude by considering structure–function relationships with outer retinal integrity and visual sensitivity, the relevance of choriocapillaris metrics as trial endpoints and stratification variables in the complement inhibitor era, and the standardization, validation and automation required before these biomarkers can be used in individual patients.

## 1. Introduction

The choroid carries the highest blood flow per unit tissue weight of any vascular bed in the human body, and its innermost layer, the choriocapillaris (CC), is the sole source of oxygen and metabolic substrate for the retinal pigment epithelium (RPE) and the photoreceptors [1]. This capillary network is not a passive conduit. Its fenestrated endothelium, its lobular organization and its close apposition to Bruch’s membrane make it a functional unit with the cells it supplies, and disturbance of any component of that unit propagates to the others [2].

That the choriocapillaris degenerates with age and in disease has been known since the morphometric studies of the 1990s, which documented progressive loss of capillary density and diameter together with thickening of Bruch’s membrane in aging donor eyes [3]. For most of the subsequent three decades, however, the layer remained largely inaccessible in vivo. Indocyanine green angiography visualizes the choroidal circulation but does not resolve the choriocapillaris as a distinct compartment, and structural optical coherence tomography (OCT) measures the thickness of the choroid without distinguishing its perfused from its stromal components. The consequence was a persistent gap between a rich histopathological literature and a clinical practice that could not act on it.

Optical coherence tomography angiography (OCTA) has closed much of that gap. Because it detects motion contrast without dye, OCTA can be repeated as often as required, and swept-source instruments operating at longer wavelengths penetrate the RPE sufficiently to render the choriocapillaris as an en face pattern of flow signal interrupted by dark regions, the flow voids or flow deficits [4], a term that denotes the absence of detectable flow signal rather than proven anatomical loss (Section 4.7). The quantification of these deficits, once methodological conventions for slab selection, shadow compensation and thresholding were established, has produced a rapidly expanding body of evidence linking choriocapillaris impairment to disease onset, progression and outcome across a wide range of chorioretinal conditions [5].

This progress has been uneven in an instructive way. The measurements are now reproducible within a given device and protocol, and longitudinal associations with clinically meaningful endpoints have been reported, although in a small number of cohorts and, for several key findings, without independent replication. Yet absolute values remain incomparable between studies that used different slabs, thresholds or compensation strategies, normative databases are limited, and the artefacts that arise precisely in diseased eyes—under drusen, beneath serous detachments, over atrophic RPE—are those most likely to distort the result. Any clinical use of these biomarkers must therefore be built on an explicit account of how they were obtained.

This review has three aims. First, to summarize the anatomy, physiology and pathobiology of the choriocapillaris in a form relevant to image interpretation. Second, to describe the imaging modalities and quantitative metrics available, with the methodological caveats attached to each. Third, to review the evidence for choriocapillaris involvement across retinal diseases, its relationship with the outer retinal structure and visual function, and its emerging role as a stratification variable and trial endpoint. Figure 1 summarizes the anatomical and pathophysiological framework on which the remainder of the review depends.

## 2. Materials and Methods

This is a narrative review, structured with reference to the SANRA scale for the assessment of narrative review articles [6]. The literature search described below served as the starting point for an expert-curated, purposive synthesis rather than as a systematic identification process; accordingly, no record counts, PRISMA flow diagram, formal risk-of-bias assessment or quantitative synthesis are reported, and the specificity of the search description should not be taken to imply a degree of procedural rigor that was not applied. PubMed/MEDLINE, Scopus and Web of Science were searched for articles published between 1 January 1990 and 31 May 2026 (final search date 31 May 2026), combining the terms “choriocapillaris”, “choroid”, “flow deficit(s)”, “flow void(s)”, “signal void”, “optical coherence tomography angiography”, “swept-source”, “signal compensation”, “indocyanine green angiography” and “choroidal vascularity index” with disease terms including “age-related macular degeneration”, “drusen”, “reticular pseudodrusen”, “geographic atrophy”, “macular neovascularization”, “pachychoroid”, “central serous chorioretinopathy”, “polypoidal choroidal vasculopathy”, “diabetic retinopathy”, “diabetic choroidopathy”, “myopia”, “retinitis pigmentosa”, “Stargardt”, “choroideremia”, “white dot syndromes”, “uveitis”, “hypertension”, “sickle cell” and “systemic sclerosis”, and with methodological terms including “repeatability”, “thresholding”, “deep learning”, “microperimetry” and “dark adaptation”. Reference lists of retrieved articles and of relevant reviews were screened manually. The search was re-run at the cut-off date before resubmission, and recent methodological, longitudinal and structure–function studies identified in this way were incorporated.

Inclusion principles were as follows: peer-reviewed original articles in humans (in vivo imaging or histopathology), randomized trials and consensus or guideline documents were eligible; conference abstracts and single case reports were not used as primary evidence. Priority was given to histopathological studies with quantitative morphometry, to longitudinal imaging cohorts, to methodological validation and repeatability studies, and to randomized trials where relevant to therapeutic implications. Cross-sectional case series were included where they constitute the only available evidence for a given condition and are identified as such. Where studies addressing the same question disagreed, both were cited and the discrepancy is discussed. Only English-language publications were considered. Because a large and active body of OCTA research, particularly on pachychoroid disease and pathological myopia, is published in East Asian languages, this restriction is a specific rather than a merely general source of selection bias and is likely to have under-represented work on those conditions. No formal risk-of-bias assessment or quantitative synthesis was undertaken, and the final selection of included studies reflects the authors’ judgment of clinical and methodological relevance; this limitation is addressed in Section 15.

No generative artificial intelligence was used to generate data or results. The authors reviewed and take full responsibility for the content of this manuscript.

## 3. Anatomy, Physiology and Pathobiology of the Choriocapillaris

### 3.1. Architecture and Regional Organization

The choriocapillaris is a single-layered, planar network of anastomosing capillaries lying immediately external to Bruch’s membrane. At the posterior pole, the capillaries are densely packed, of large caliber relative to systemic capillaries, and organized into functional lobules each supplied by a terminal choroidal arteriole and drained peripherally by venules [2]. The density of this network is greatest submacularly and decreases towards the periphery, where the pattern becomes progressively more reticular and the intercapillary spaces widen; en face OCTA images acquired at different eccentricities reproduce this gradient and match scanning electron microscopy of corrosion casts [7].

Two features determine the appearance of the layer on OCTA. The endothelium is fenestrated on the aspect facing Bruch’s membrane, which permits the free exchange required by the RPE and underlies the leakage seen on fluorescein angiography when the RPE barrier fails. And the intercapillary spacing at the posterior pole is close to the transverse resolution limit of current OCTA instruments, which means that in healthy eyes, some proportion of the dark regions on an en face choriocapillaris image represents normal intercapillary tissue rather than absent flow—a point with direct consequences for quantification [8].

### 3.2. The RPE–Bruch’s Membrane–Choriocapillaris Complex

The functional interdependence of this three-part complex is best illustrated by the vascular endothelial growth factor (VEGF) axis. The RPE secretes VEGF in a polarized fashion, predominantly basally towards the choroid, and VEGF receptors are concentrated on the inner aspect of the choriocapillaris endothelium [9]. This constitutive basal secretion is not merely permissive but necessary: conditional ablation of RPE-derived soluble VEGF in animal models produces rapid choriocapillaris atrophy followed by photoreceptor loss [10]. The choriocapillaris is therefore trophically dependent on the RPE, just as the RPE is metabolically dependent on the choriocapillaris.

This bidirectional dependency means that the causal direction of any observed association is rarely self-evident. Choriocapillaris loss may follow RPE dysfunction, precede it, or the two may deteriorate in parallel under a shared insult. The question matters therapeutically, and the imaging literature reviewed below has been able to address it only partially, largely through the study of eyes at risk before overt disease appears.

### 3.3. Mechanisms of Choriocapillaris Dysfunction

Several mechanisms, not mutually exclusive, have been implicated. *Complement-mediated endothelial injury* is the best characterized. The membrane attack complex accumulates in the choriocapillaris with age and is markedly increased in eyes with age-related macular degeneration (AMD), where its deposition correlates with capillary loss and with high-risk complement genotypes [11]. This provides a coherent link between the genetic architecture of AMD and its earliest anatomical lesion, and it constitutes the rationale for complement-directed therapy discussed in Section 14 [12].

*Bruch’s membrane thickening and lipid accumulation* increase the diffusion distance between the capillary lumen and RPE and reduce hydraulic conductivity, compounding any reduction in perfusion [3,13]. *Loss of trophic support* follows RPE dysfunction through the VEGF mechanism described above. *Oxidative stress and inflammation* affect the endothelium and RPE alike. Finally, *venous overload* has been proposed as the unifying mechanism of the pachychoroid spectrum: congestion of the choroidal venous outflow with dilatation of Haller layer vessels mechanically compresses the overlying choriocapillaris, producing flow deficits in a layer that may not be intrinsically diseased [14].

The temporal sequence has been examined histologically. Ultrastructural work in donor eyes found choriocapillaris breakdown, with endothelial cell loss and basement membrane changes, in regions where the overlying RPE and photoreceptors were still comparatively preserved, supporting a primary or at least early vascular contribution in AMD [15]. Histological series are, however, necessarily cross-sectional, and cannot establish the sequence of events within any individual eye. Consistent with this, choriocapillaris density in early AMD donor eyes decreases in proportion to overlying drusen burden but is also reduced in areas without drusen [16].

## 4. Imaging the Choriocapillaris

No single modality captures the choriocapillaris completely. The modalities in current use are summarized in Table 1 and discussed below in order of the information they contribute; comprehensive technical reviews of choroidal OCT angiography are available elsewhere [17,18].

### 4.1. Indocyanine Green Angiography

Indocyanine green angiography (ICGA) remains the reference for choroidal vascular filling. Its near infrared fluorescence traverses the RPE, and the strong protein binding of the dye limits extravasation from the fenestrated choriocapillaris endothelium, so that filling delays, watershed zones, hyperpermeability and polypoidal lesions are demonstrated with a clarity no other technique matches. ICGA is dynamic, providing temporal information that OCTA cannot. Its limitations are equally clear: it is invasive, it cannot readily be repeated, as an en face fluorescence technique it provides no depth resolution to separate the choriocapillaris from deeper choroidal layers, and its interpretation is qualitative in most clinical settings.

### 4.2. Structural OCT: Choroidal Thickness and the Choroidal Vascularity Index

Enhanced-depth imaging and swept-source OCT permit the measurement of choroidal thickness, which is abnormally increased in the pachychoroid spectrum and reduced in advanced AMD, myopia and some inherited dystrophies. Thickness, however, aggregates the lumen and stroma and varies with age, axial length, refraction, time of day and systemic hemodynamics, which limits its discriminatory value. The choroidal vascularity index, defined as the ratio of luminal to total choroidal area on binarized cross-sectional images, was introduced to address this and is less sensitive to axial length and diurnal variation [19]. Neither metric isolates the choriocapillaris, and both should be read as descriptors of the choroid as a whole.

### 4.3. OCT Angiography: Slab Definition and Signal Compensation

OCTA is the only technique that images the choriocapillaris as a distinct layer in vivo, and the validity of any measurement depends on three sequential decisions. The first is the *slab*. The physiologically defensible definition extends from the outer boundary of Bruch’s membrane to approximately 20 μm beneath it [7]. Slabs that begin too anteriorly incorporate the RPE signal and projection artefact; slabs that extend too posteriorly include Sattler layer vessels, which have different flow characteristics and produce systematically different flow deficit values. Direct comparison of slab choices in the same eyes demonstrated that this decision alone substantially alters the quantified deficit [20].

The second is *compensation*. Because the RPE attenuates the beam, any variation in RPE reflectivity—pigment clumping, drusen, atrophy—modulates the signal reaching the choriocapillaris and creates dark regions that mimic absent flow. Compensation uses the structural information from the same slab to correct the angiographic signal, and its validation was a prerequisite for meaningful quantification [7]. The third is *binarization*. Converting a grayscale flow image into a binary map of perfused and non-perfused pixels requires a threshold, and thresholds derived individually from each image are not comparable across eyes; thresholds derived from a normal database are preferable [7]. Reported flow deficit values are exquisitely sensitive to this choice, as well as to any prior brightness or contrast adjustment [21].

### 4.4. Quantitative Metrics

Three families of metrics are in use. The *flow deficit percentage* (FD%) is the proportion of the analyzed area occupied by deficits and is the most widely reported. The *mean flow deficit size* and the *flow deficit density* describe whether a given FD% arises from many small deficits or from fewer large ones, a distinction with biological meaning since only deficits larger than normal intercapillary spacing plausibly represent true dropout; excluding smaller deficits improves the robustness of the estimate [8]. Finally, the *statistical distribution* of deficit sizes has been shown to follow a power law whose parameters vary with age and disease, offering a description of the layer that is independent of any single threshold [4].

Repeatability is good under controlled conditions and improves further with registration and averaging of multiple scans, which raises signal-to-noise and reduces the influence of individual acquisition artefacts [22,23]. Averaging costs acquisition time and is not always practical in elderly patients with unstable fixation, which is a recurring tension in this literature: the techniques that improve measurement quality are least applicable in the eyes where measurement matters most. Scan size introduces a further consideration, since 3 × 3 mm scans sample the choriocapillaris at higher transverse resolution than 6 × 6 mm scans of the same instrument, and values obtained at the two settings are not interchangeable [24].

### 4.5. Artefacts and Pitfalls

Several artefacts arise specifically in the eyes of interest. Drusen attenuate and displace the overlying signal and require compensation adjusted for their presence; without it, flow deficits are systematically overestimated beneath them. Subretinal and sub-RPE fluid displace the choriocapillaris posteriorly and attenuate the signal, so that reduced flow beneath a serous detachment may be artefactual rather than real. Areas of RPE atrophy produce the opposite effect, increasing signal transmission and potentially masking genuine deficits. Media opacity, low signal strength, eye movement and segmentation failure all degrade the measurement, and in advanced disease, segmentation of Bruch’s membrane itself may fail. Published consensus guidance addresses each of these and should be regarded as the minimum methodological standard for any study reporting choriocapillaris quantification [5].

### 4.6. The Standardization Problem

The cumulative effect of these choices is that absolute flow deficit values cannot be transferred between studies, devices or software versions. A reported FD% is interpretable only in conjunction with the slab definition, compensation method, threshold derivation, scan size, averaging strategy and exclusion criteria used to obtain it. Until cross-platform normative databases and agreed processing conventions exist, the defensible uses of these metrics are comparison between eyes imaged under identical conditions and change over time within the same eye and instrument. This constraint recurs throughout the clinical sections that follow and is the principal obstacle to individual-patient application.

### 4.7. Terminology and Levels of Inference

The terms used in this literature carry different levels of inference, and they are used here accordingly. A flow deficit (or flow void, the term preferred in the earlier literature) is a measurement: a region of the compensated, binarized en face choriocapillaris angiogram in which the decorrelation signal falls below the detection threshold of the instrument and processing pipeline. It may reflect the true absence of perfusion, but it may equally reflect flow that is too slow to be resolved within the interscan time, attenuation of the signal by overlying tissue, segmentation error or other acquisition-related artefact. Non-perfusion, capillary dropout and capillary loss are anatomical inferences, and they require corroboration: histology in donor eyes, persistence across repeated compensated acquisitions, or spatial correspondence with independent structural evidence. Throughout this review, “flow deficit” is therefore used when reporting OCTA measurements; “dropout” and “capillary loss” are reserved for histological findings or for OCTA findings whose anatomical interpretation is supported by such corroboration; and “impairment” is used as a neutral term where the level of inference is uncertain. Where a cited study used “non-perfusion” in its own title or terminology, its finding is reported here as a flow deficit unless the authors provided evidence of anatomical loss.

## 5. The Normal and Aging Choriocapillaris

Any disease-related change must be read against a moving baseline. Flow deficits increase with age in normal eyes: in a swept-source OCTA study of healthy subjects across the adult age range, both the percentage and the mean size of macular choriocapillaris flow deficits rose significantly with advancing age, with the central macula most affected [25]. This mirrors the histological finding of progressive capillary loss and luminal narrowing in aging donor eyes [3] and establishes that age-matched comparison is mandatory.

Topography also matters. Widefield swept-source OCTA mapping of healthy eyes demonstrated a systematic increase in flow deficits from the posterior pole towards the periphery, consistent with the anatomical gradient in capillary density [26]. Consequently, the analyzed region must be specified and matched: an FD% derived from a 3 × 3 mm macular scan is not comparable with one derived from a widefield montage, and regional analyses should be reported by sector rather than as a single global figure. Sex, axial length, systemic blood pressure and smoking status have all been reported to influence measurements to varying degrees, and their inconsistent adjustment across studies contributes to the heterogeneity of published normative values.

## 6. Age-Related Macular Degeneration

AMD is the condition in which choriocapillaris imaging has been most extensively applied, and the one in which its prognostic value is best supported, although still by a small number of cohorts.

### 6.1. Early and Intermediate AMD

Donor eye morphometry demonstrated that choriocapillaris density in early AMD is reduced in proportion to overlying drusen burden, but also, importantly, in regions free of drusen, indicating that vascular loss is not simply a consequence of overlying deposit [16]. In vivo studies have reproduced and extended this. Flow deficits are increased in eyes with early and intermediate AMD relative to age-matched controls, and although the impairment is greatest beneath and immediately around drusen, it is not confined to their vicinity [27].

The prognostically relevant observation is longitudinal. In a retrospective cohort, choriocapillaris flow impairment at baseline was associated with both the subsequent development of new drusen and the enlargement of existing ones, in regions that appeared normal at the time of the initial scan [28]. This specific finding, with drusen as the endpoint, has not yet been replicated in an independent cohort. Converging evidence does, however, support the broader association between baseline choriocapillaris impairment and progression: greater flow deficits have been reported in intermediate AMD eyes that subsequently progressed to advanced disease [29]; flow deficits increase measurably over 12 months even in clinically stable intermediate AMD [30]; a recent 24-month cohort found a greater baseline flow deficit area in eyes that later developed geographic atrophy, although not in those that developed neovascular disease [31]; and progressive choriocapillaris change accompanies stage progression across the AMD spectrum [32]. Taken together, these studies establish a temporal association between choriocapillaris impairment and the visible lesions of AMD, and they identify the layer as a candidate target for intervention before irreversible outer retinal damage occurs. They do not establish that choriocapillaris dysfunction is the initiating event. Because the RPE, Bruch’s membrane and choriocapillaris form an interdependent unit (Section 3.2), subclinical RPE or Bruch’s membrane change preceding both the flow deficit and the drusen cannot be excluded by imaging alone, and temporal precedence on OCTA should be distinguished from causal inference.

### 6.2. Subretinal Drusenoid Deposits

Subretinal drusenoid deposits, also termed reticular pseudodrusen, are associated with particularly marked choriocapillaris compromise. Eyes with these deposits show greater flow deficits than eyes with conventional drusen, and the extent of flow deficits correlates with poorer visual acuity [33,34]. The association is mechanistically coherent, since the topographic distribution of subretinal drusenoid deposits corresponds to regions of high rod density and elevated metabolic demand, and it helps explain why these deposits confer a higher risk of progression to both atrophic and neovascular disease. The caveat is that the overlying deposits themselves attenuate a signal, so that a proportion of the measured deficit may be artefactual unless compensation is carefully applied.

### 6.3. Geographic Atrophy

Within areas of geographic atrophy (GA), choriocapillaris flow is severely reduced or absent, which is expected given the loss of the overlying RPE and its trophic support. The clinically informative finding concerns the retina *surrounding* the atrophy. Flow deficits in the junctional zone are increased relative to more distant retina, and their magnitude correlates with the subsequent rate of lesion enlargement [35,36]. In one analysis, flow deficits within approximately 500 μm of the lesion margin predicted the yearly enlargement rate, whereas deficits further away did not [37]; in another, deficits across the entire scan area correlated more strongly with growth than those immediately adjacent [35]. Local analyses relating growth trajectories to spatially matched flow deficits have since confirmed the association while showing that its strength varies regionally [38], and independent cohorts have reported that choriocapillaris impairment predicts lesion enlargement [39].

The discrepancies between these studies are informative rather than disqualifying: they reflect differences in instrument, scan size, compensation strategy and the definition of the perilesional region, which is precisely the standardization problem set out in Section 4.6. What is consistent across all of them is the direction of the association. Choriocapillaris flow deficits surrounding GA constitute a risk marker for progression, and they are therefore a candidate stratification variable for trials in which the enlargement rate is the primary endpoint.

### 6.4. Macular Neovascularization

Type 1 macular neovascularization arises from the choriocapillaris and grows in the sub-RPE space, and the hypothesis that localized choriocapillaris ischemia drives this process is long-standing. OCTA has shown that flow deficits are increased in the choriocapillaris adjacent to neovascular lesions and, more informatively, that non-exudative type 1 neovascularization can be detected in eyes before any exudation occurs. Standardized terminology for reporting these findings has been established by consensus and should be used [40]. The relationship between choriocapillaris impairment and neovascular growth is plausibly reciprocal, since established neovascularization alters flow in the surrounding capillary bed, and cross-sectional data cannot resolve the direction.

### 6.5. Fellow Eyes and Eyes at Risk

Study of the unaffected fellow eyes of patients with unilateral neovascular AMD isolates the choriocapillaris from the confounding effects of established macular disease. Such eyes show increased flow deficits and a higher prevalence of subclinical neovascularization relative to controls [41], and more recent work using signal-compensated swept-source acquisition has confirmed early choriocapillaris dysfunction in this population [42]. Because these eyes are at high risk of conversion, they represent the most efficient setting in which to test whether choriocapillaris metrics add predictive value beyond established structural risk factors, and whether they are modifiable. The choriocapillaris findings across the principal chorioretinal diseases discussed in this review are summarized in Table 2.

## 7. The Pachychoroid Spectrum and Central Serous Chorioretinopathy

The pachychoroid spectrum comprises pachychoroid pigment epitheliopathy, central serous chorioretinopathy (CSC), pachychoroid neovasculopathy and polypoidal choroidal vasculopathy, unified by an abnormally thickened choroid with dilated outer choroidal vessels [43,44]. En face swept-source imaging established that these dilated Haller layer vessels, the so-called pachyvessels, occupy the full choroidal thickness and terminate abruptly, and that they correspond spatially to regions of increased choroidal thickness [43].

The choriocapillaris in these eyes shows increased flow deficits, characteristically overlying the pachyvessels [45,46]. The mechanistic interpretation proposed for this finding differs fundamentally from that in AMD: rather than a primary capillary degeneration, the flow deficits are thought to reflect the mechanical compression and attenuation of the choriocapillaris by the dilated vessels beneath, with secondary ischemia and impairment of the RPE barrier proposed to lead to subretinal fluid accumulation. The venous overload hypothesis provides a coherent explanatory framework in which choroidal venous congestion, rather than arterial insufficiency, is postulated as the initiating event [14]. It should be emphasized that these mechanisms remain inferential: the spatial coincidence of flow deficits with pachyvessels is consistent with compression but does not demonstrate it, direct measurements of choroidal venous pressure in patients are not available, and the OCTA appearance of a compressed capillary is not distinguishable from that of a degenerated one. Widefield OCTA has extended these observations beyond the posterior pole, demonstrating that choroidal thickening and choriocapillaris alteration in CSC involve regions well outside the macula and are detectable in clinically unaffected fellow eyes [47].

Two caveats deserve emphasis. First, the artefact problem is at its most acute here: subretinal fluid and pigment epithelial detachments attenuate the signal and displace the slab, so that flow deficits measured beneath active detachments may be substantially artefactual. Studies that explicitly excluded regions affected by fluid and RPE alterations have reported more conservative estimates of true choriocapillaris impairment. Second, the appearance evolves with disease phase, so acute, chronic and resolved CSC should not be pooled. Whether choriocapillaris metrics can predict which eyes will develop chronic disease or secondary neovascularization is an open question of clear clinical relevance.

## 8. Diabetic Retinopathy and Diabetic Choroidopathy

The retinal microvasculature has dominated the diabetic imaging literature, but diabetic choroidopathy is a well-described histopathological entity comprising capillary dropout, microaneurysm formation and basement membrane thickening in the choriocapillaris. OCTA studies have confirmed increased choriocapillaris flow deficits in diabetic eyes, present even before clinically visible retinopathy and increasing with retinopathy severity [48,49]. This preclinical signal is not, however, a universal finding: a recent study using projection-resolved OCTA with automated exclusion of shadowing artefacts and of areas beneath retinal fluid found flow deficits to be increased in eyes with non-proliferative and proliferative retinopathy, but not in diabetic eyes without retinopathy [50], which suggests that part of the earlier preclinical signal may be attributable to artefact or to methodological differences. Reduced decorrelation signal in the inner choroid has been associated with the presence of diabetic macular edema and with structural outer retinal change [51]. More recently, choriocapillaris flow area in diabetic eyes has been correlated with photoreceptor outer segment length and with glycemic control and systolic blood pressure, linking the vascular finding both to the outer retinal structure and to systemic exposure [52].

Two clinical implications follow. Choriocapillaris compromise offers a mechanistic explanation for outer retinal abnormalities in diabetes, including ellipsoid zone disruption, that are not fully accounted for by retinal capillary non-perfusion; and it may contribute to the incomplete functional recovery observed in some eyes after anatomically successful treatment of macular edema. The evidence remains predominantly cross-sectional, and longitudinal data establishing whether baseline choriocapillaris metrics predict progression or treatment response in diabetic eyes are limited.

## 9. Myopia and Myopic Maculopathy

Choroidal thinning is the defining structural feature of pathological myopia, and the choriocapillaris participates in this process. Quantitative OCTA has demonstrated reduced choriocapillaris flow signal in myopic eyes, with the deficit increasing with axial length [53]. Choriocapillaris attenuation is thought to contribute to the development of the atrophic lesions that define the higher categories of the international classification of myopic maculopathy, and to the pathogenesis of myopic neovascularization [54].

Quantification in these eyes carries a specific technical problem. Ocular magnification varies with axial length, so that a nominal 3 × 3 mm scan subtends a larger retinal area in a long eye than in a short one. Unless magnification correction is applied, comparisons between myopic and emmetropic eyes, and between myopic eyes of differing axial length, are systematically biased. Not all published studies report such correction, which is a plausible source of the heterogeneity in this literature.

## 10. Inherited Retinal Diseases

In retinitis pigmentosa, OCTA has demonstrated reduced choriocapillaris flow signal alongside retinal capillary rarefaction, with the degree of impairment relating to the extent of outer retinal loss [55]. Whether choriocapillaris attenuation is a primary component of the disease or a secondary consequence of photoreceptor and RPE degeneration, with loss of trophic support, remains unresolved; the developmental and mechanistic evidence favors the latter, but a contributory vascular role cannot be excluded.

In Stargardt disease, choriocapillaris flow deficits accompany the RPE atrophy characteristic of the condition and extend somewhat beyond its visible boundaries [56]. Choroideremia offers the most instructive case, since the causative gene is expressed in the RPE, photoreceptors and choroid alike. OCTA studies have shown choriocapillaris flow deficits closely spatially correlated with overlying degeneration, with a sharp transition at the border of the preserved retina, consistent with the layers failing together rather than sequentially [57]. As gene and cell therapies for these conditions advance, the status of the choriocapillaris becomes practically relevant, since a treatment directed at the photoreceptors or RPE may be futile in areas where the supplying vasculature has already been lost.

## 11. Inflammatory Chorioretinopathies

White dot syndromes have been substantially reinterpreted through choriocapillaris imaging. In placoid chorioretinitis, OCTA demonstrates well-demarcated areas of choriocapillaris flow reduction corresponding to the hypofluorescent lesions on ICGA, supporting a primary choriocapillary inflammatory occlusion rather than a primary RPE process [58]. Serial imaging in acute posterior multifocal placoid pigment epitheliopathy has shown flow deficits appearing with the acute lesions and partially reperfusing during resolution, with the degree of residual flow deficit corresponding to the extent of permanent outer retinal damage [59].

Comparable findings have been reported in multifocal choroiditis and punctate inner choroidopathy, where flow deficits colocalize with active lesions [60], and in Vogt–Koyanagi–Harada disease, where choriocapillaris impairment accompanies the acute exudative phase and improves with treatment [61]. Quantitative flow deficit metrics have also been applied to uveitic eyes using the same automated identification pipeline developed for AMD, which makes serial comparison within a patient feasible [62]. The practical value here is greater than in the degenerative diseases: because these conditions are treatable and their activity fluctuates, a non-invasive measure of choriocapillary perfusion that can be repeated at every visit is a genuine addition to the clinical assessment, and its use for monitoring the response to immunosuppression is a reasonable extension of the current evidence.

## 12. Systemic Disease and Oculomics

The choriocapillaris is a systemic vascular bed and reflects systemic disease. Increased flow deficits have been documented in mitochondrial disease and in pseudoxanthoma elasticum, where Bruch’s membrane calcification alters the interface with the capillary layer [63]. Systemic hypertension is associated with altered ocular microvascular perfusion [64], and the broader oculomics literature has shown that retinal and choroidal imaging carries information about cardiovascular and renal status [65]. Other systemic vascular conditions relevant to ophthalmology have been examined less thoroughly at the level of the choriocapillaris. In sickle cell disease, OCTA has documented macular flow loss and reduced vessel density in the retinal capillary plexuses, corresponding to the temporal macular thinning characteristic of sickle cell maculopathy [66]; choriocapillaris-specific quantification in this condition is so far limited to preliminary reports, although choriocapillaris occlusion has been proposed to contribute to the retinopathy. In systemic sclerosis, reduced choriocapillaris perfusion metrics have been reported in the absence of clinical retinopathy [67,68], including in very early disease, where they correlated with nailfold capillaroscopy findings in keeping with the vascular hypothesis of that condition [67]. These studies used spectral domain instruments and vessel density or flow area metrics rather than compensated flow deficit quantification, so their values are not comparable with those reported in AMD, but they illustrate that the choriocapillaris is accessible as a window on systemic microvascular disease.

The interpretive implication for the disease-specific sections above is that systemic vascular comorbidity is a confounder in every study of choriocapillaris flow. Hypertension, diabetes, smoking and cardiovascular disease are common in the elderly populations in which AMD is studied, and their adjustment has been inconsistent. The opposite framing is also worth noting: if choriocapillaris metrics reflect systemic vascular health, they may have value beyond ophthalmology, although this remains speculative.

## 13. Structure–Function Relationships

The clinical significance of a choriocapillaris biomarker depends on its relationship with vision, and it is useful to separate two kinds of evidence: associations between flow deficits and structural markers of outer retinal damage, which are indirect, and associations with measured visual function, which are the evidence that matters for biomarker qualification.

### 13.1. Structural Correlates

Regions of flow deficit correspond topographically to disruption of the ellipsoid zone and to attenuation of the external limiting membrane, the OCT signatures of photoreceptor compromise. In GA, photoreceptor degeneration extends beyond the boundaries of visible atrophy and, in one analysis, progressed in a manner that paralleled the distribution of choriocapillaris impairment [69]; this observation rests on a single cohort and has not yet been replicated. In intermediate AMD, flow deficits are greatest beneath and around drusen but extend into drusen-free retina [27], and in diabetic eyes, they have been related to photoreceptor outer segment length [52]. Structural correspondence of this kind is consistent with, but does not demonstrate, a functional consequence.

### 13.2. Functional Correlates

Visual acuity is an insensitive endpoint for a process that begins outside the fovea, although even acuity has shown an association: flow deficits in eyes with subretinal drusenoid deposits correlate with reduced visual acuity [33]. More informative are the tests matched to the predominantly rod-mediated dysfunction of early outer retinal disease. In early and intermediate AMD, the flow deficit percentage on compensated, averaged swept-source OCTA correlated with scotopic microperimetric sensitivity, both in the central 10° and across an 18° grid, after adjustment for the presence of subretinal drusenoid deposits [70]; in the same population, baseline inner-choroid flow deficits and scotopic sensitivity were reported as predictors of progression to incomplete RPE and outer retinal atrophy over 24 months [71]. In the ALSTAR2 cohort, a greater flow deficit percentage was associated with delayed rod-mediated dark adaptation and with worse performance on most other visual function tests, although not on photopic or mesopic light sensitivity, and dark adaptation was the only function that already separated early AMD from normal aging [72]. In GA, spatially matched analysis showed that choriocapillaris flow deficit and retinal sensitivity on microperimetry were inversely correlated, and that both improved with distance from the atrophy margin, indicating functional compromise beyond the visible lesion [73]. These studies are cross-sectional or short-term, single-center and modest in size, and they used different instruments (including spectral domain devices in the ALSTAR2 analysis), so the strength of the association is not transferable between them; but they are consistent in direction, and they establish that choriocapillaris flow deficits have a measurable functional correlate at the level of individual retinal loci, and not only a structural one.

The combination of spatially resolved flow mapping with microperimetry, dark adaptation and low-luminance acuity therefore remains the natural design for biomarker qualification, and its extension into longitudinal, multicenter settings is among the more valuable directions for the field. The intermediate anatomical endpoints defined by consensus—incomplete and complete RPE and outer retinal atrophy—provide a useful bridge, since they are reproducible and occur earlier than measurable acuity loss [74,75].

## 14. Therapeutic and Clinical Implications

### 14.1. Complement Inhibition and the Choriocapillaris

The approval of complement inhibitors for GA has made choriocapillaris biology therapeutically relevant. Pegcetacoplan, a C3 inhibitor, reduced the growth of GA lesions in a phase 2 trial and in the phase 3 OAKS and DERBY trials [76,77], and avacincaptad pegol, a C5 inhibitor, produced a comparable anatomical effect in GATHER2 [78]. In both programs, the reduction in lesion growth was not accompanied by a demonstrable visual benefit within the trial period, and new-onset exudative neovascularization was more frequent in treated eyes.

Two points connect this to the present review. Mechanistically, the membrane attack complex accumulates in the choriocapillaris rather than in the retina [11], so complement inhibition is plausibly acting, at least in part, on the vascular layer; a post hoc analysis reported reduced photoreceptor degeneration beyond the areas of atrophy in treated eyes, consistent with an effect on the tissue surrounding the lesion [79]. Practically, the discrepancy between anatomical and functional outcomes is exactly the problem that better biomarkers might address. Here, it is necessary to separate what has been shown from what is proposed. What has been shown is that perilesional choriocapillaris flow deficits are associated with the natural rate of lesion enlargement [35,37,39]. What has not been shown is that they predict the response to complement inhibition: no trial has stratified patients by choriocapillaris metrics or reported them as an outcome, and a biomarker of natural progression does not necessarily identify treatment responders, since the eyes that progress fastest may be those in which the vascular damage is least reversible. The use of perilesional flow deficits to select patients for complement inhibition, or as a mechanistic endpoint closer to the drug’s presumed site of action than lesion area, is therefore a hypothesis for prospective testing rather than a current application.

### 14.2. Other Therapeutic Contexts

In CSC, half-dose photodynamic therapy is effective for chronic disease [80], and it acts on the choroidal vasculature; whether pre-treatment choriocapillaris metrics predict response, and whether treatment normalizes them, are questions well suited to non-invasive serial imaging. In neovascular AMD, the effect of repeated anti-VEGF administration on a capillary bed that is trophically dependent on VEGF is a legitimate concern, but the direct longitudinal evidence is limited and inconsistent. One study reported a reduction in choriocapillaris vessel density during anti-VEGF therapy [81], whereas others have described partial reperfusion of the choriocapillaris surrounding the neovascular lesion after a loading phase of aflibercept, ranibizumab or faricimab, which the authors attributed to regression of the lesion and resolution of exudation rather than to a direct effect of the drug on the capillary bed [82,83]. These studies differ in instrument, metric, region analyzed and duration, and none separates the effect of the drug from that of the disease and of treatment-related structural change. The question therefore remains unresolved, and longitudinal choriocapillaris measurement offers a means of addressing it that did not previously exist. In inflammatory chorioretinopathies, as noted above, reperfusion can be documented directly and used to guide immunosuppression.

### 14.3. Automation and Artificial Intelligence

Every methodological step described in Section 4 and summarized in Figure 2—segmentation of Bruch’s membrane, compensation, thresholding, artefact detection, regional analysis—is a candidate for automation, and deep learning has been applied successfully to comparable segmentation problems in retinal imaging [84]. Choriocapillaris-specific work is more limited than the general enthusiasm for artificial intelligence in the retina might suggest. The validated compensation and thresholding pipelines already in use are largely automated within a given laboratory [7,8]; automated flow deficit identification has been applied outside AMD, for example, to uveitis [62]; and a recent projection-resolved OCTA method incorporated automated detection of shadowing from hard exudates, retinal vessels and vitreous floaters, and of areas beneath retinal fluid, before quantifying flow deficits in diabetic eyes, with high test–retest repeatability [50]. What does not yet exist is a cross-device, externally validated, learning-based pipeline for choriocapillaris quantification, or a published deep learning model for choriocapillaris artefact detection comparable to those available for retinal fluid or geographic atrophy. Self-supervised foundation models trained on large unlabeled imaging datasets offer a plausible route to this with fewer expert annotations than supervised approaches require [85], but this remains a direction for future work rather than a solution to the current reproducibility problem. Two caveats apply. First, automating a measurement does not validate it: an algorithm trained to reproduce a particular laboratory’s thresholding convention will reproduce its biases faithfully and at scale. Second, the computational and annotation bottleneck is not the only practical obstacle. Meaningful choriocapillaris quantification currently requires swept-source instrumentation with validated compensation, which is considerably less widely available than the spectral domain OCT on which most clinical practice depends; spectral domain angiography can image the layer, as several of the studies cited here did, but with greater attenuation and less comparable values (Table 1). This hardware barrier is a separate and equally real obstacle to the individual-patient applicability called for in the Conclusions. The minimum methodological requirements for reporting quantitative choriocapillaris measurements, and the consequence of omitting each, are summarized in Table 3.

## 15. Limitations and Future Directions

This review is narrative rather than systematic: the search was restricted to three databases and to English-language publications, which is a specific concern given the size of the East Asian literature on OCTA, pachychoroid disease and myopia; no formal risk-of-bias assessment was undertaken; and the selection of studies reflects the authors’ judgment (Section 2). The literature reviewed carries its own limitations, which are more consequential. Most studies are cross-sectional, retrospective and single-center; sample sizes are typically modest; several of the most cited findings, including the association of baseline flow deficits with subsequent drusen development [28] and the parallel between photoreceptor degeneration and choriocapillaris impairment in GA [69], rest on single cohorts; and the methodological heterogeneity documented in Section 4 means that numerical values cited from different studies are not directly comparable. Publication bias cannot be excluded, since negative findings in a field driven by new metrics may be under-reported. The search did identify some null and discordant results, and these are reported in the relevant sections rather than omitted: the absence of increased flow deficits in diabetic eyes without retinopathy once shadowing artefacts were excluded [50], contrasting with earlier reports [48,49]; the absence of a baseline difference in intermediate AMD eyes that later developed neovascular rather than atrophic disease [31]; reduction versus reperfusion of the choriocapillaris during anti-VEGF therapy [81,82,83]; and the divergent perilesional versus whole-scan correlations with GA growth [35,37]. Their number is small relative to the volume of positive associations, which is itself consistent with under-reporting rather than with an absence of negative results.

Several priorities follow. Standardization is the precondition for everything else: agreed slab definitions, compensation and thresholding conventions, a shared terminology that separates measured flow deficits from inferred capillary loss (Section 4.7), mandatory reporting of acquisition parameters, and cross-platform normative databases stratified by age, axial length and ethnicity. Longitudinal multicenter cohorts with harmonized protocols are needed to establish whether choriocapillaris metrics add predictive value beyond established structural and clinical risk factors, which is the question that determines clinical utility. Structure–function studies pairing spatially resolved flow mapping with microperimetry and dark adaptation would establish whether flow deficits are functionally consequential locus by locus. Automated, validated analysis pipelines are required to make any of this deployable. Finally, integration with genotype, particularly complement pathway variants, and with systemic vascular data may allow the identification of patients in whom choriocapillary compromise is the dominant mechanism and who are therefore the appropriate targets for vascular- or complement-directed intervention.

## 16. Conclusions

The choriocapillaris has moved within a decade from a layer inferred from donor eye histology to one that can be measured repeatedly in living patients. The resulting evidence is consistent in direction across a wide range of conditions: flow deficits increase with age, increase further in disease, and in age-related macular degeneration are temporally associated with, and in a small number of cohorts have predicted, subsequent drusen formation and the rate of geographic atrophy enlargement, without this establishing that the vascular change is the initiating event. In the pachychoroid spectrum, the same measurement is thought to reflect mechanical compression rather than primary capillary loss, a proposed mechanism that remains inferential but a reminder that a biomarker’s meaning is disease-specific. Across diabetic, myopic, dystrophic and inflammatory disease, choriocapillaris assessment offers a plausible mechanistic account of outer retinal damage that retinal imaging alone does not provide.

What is not yet established is individual-patient applicability. Absolute values remain incomparable across studies and devices, normative data are incomplete, the artefacts are greatest in precisely the eyes of clinical interest, and prospective demonstration that choriocapillaris metrics change management or outcome is lacking. The obstacles are practical as well as computational, since the swept-source instrumentation and validated processing on which meaningful quantification depends are not yet widely available. These are tractable problems, and the arrival of therapies plausibly acting on this vascular bed makes their solution urgent. The choriocapillaris has the potential to become a clinically actionable biomarker in chorioretinal disease, provided the field invests in the standardization and prospective validation that its measurement now requires.

## Figures and Tables

**Figure 1:**
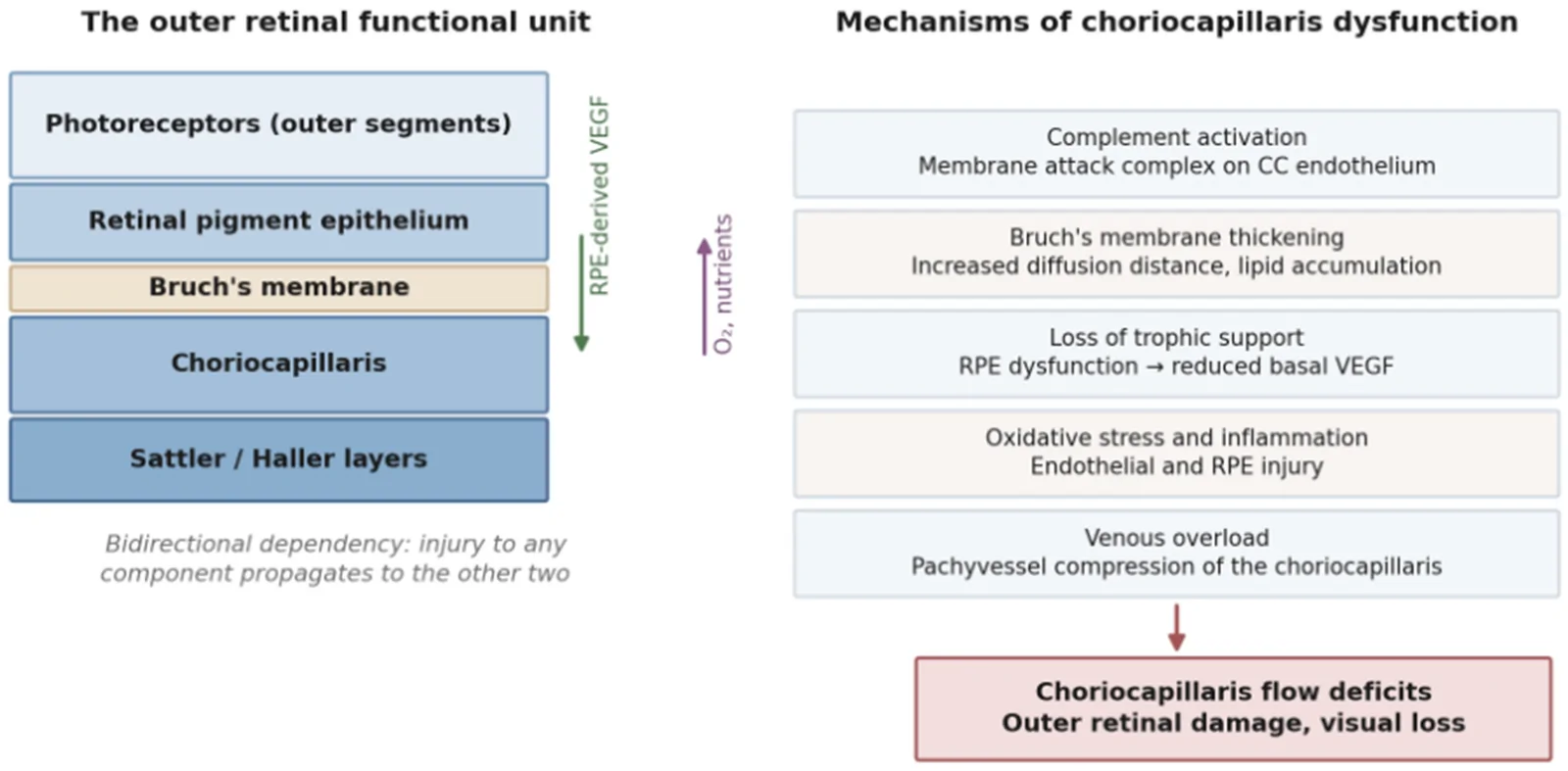
The RPE–Bruch’s membrane–choriocapillaris complex and the principal mechanisms of choriocapillaris dysfunction. The choriocapillaris supplies the outer retina across Bruch’s membrane and is in turn trophically dependent on basally secreted RPE-derived VEGF; injury to any component propagates to the others. Green arrow, basal secretion of RPE-derived VEGF towards the choriocapillaris; purple arrow, supply of oxygen and nutrients from the choriocapillaris to the outer retina; red arrow, convergence of the individual mechanisms on flow deficits and outer retinal damage. CC, choriocapillaris; RPE, retinal pigment epithelium; VEGF, vascular endothelial growth factor.

**Figure 2:**
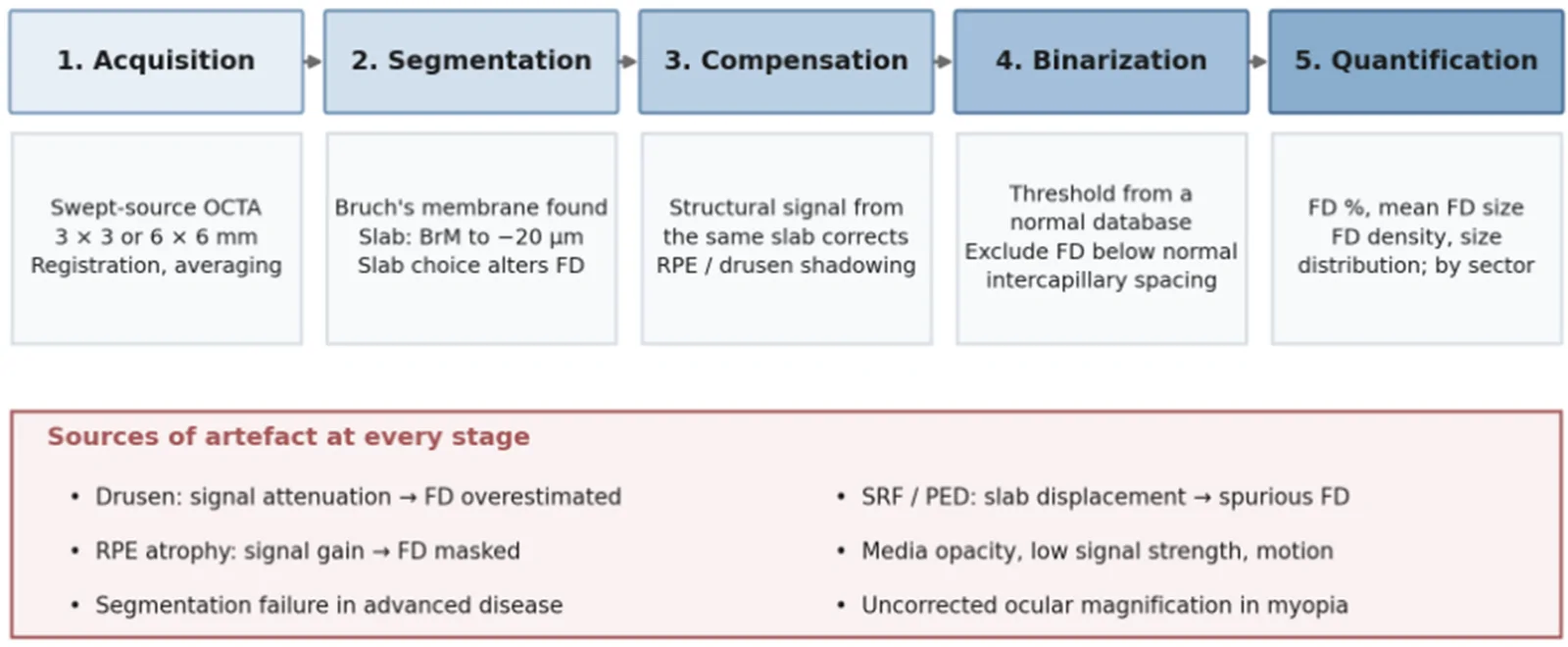
Workflow for quantitative choriocapillaris assessment with OCT angiography, showing the sequential processing decisions that determine the reported flow deficit value and the principal sources of artefact at each stage. Horizontal arrows indicate the sequence of processing steps; arrows within the lower panel indicate the effect of each artefact on the measured flow deficit. BrM, Bruch’s membrane; FD, flow deficit; OCTA, optical coherence tomography angiography; PED, pigment epithelial detachment; RPE, retinal pigment epithelium; SRF, subretinal fluid.

**Table 1:** Modalities available for assessing the choriocapillaris and the inner choroid, with their principal contributions and limitations.

Modality	What it Assesses	Principal Strengths	Principal Limitations
Indocyanine green angiography	Dynamic filling of the choroidal circulation; hyperpermeability; polypoidal lesions	Temporal information; penetrates the RPE; reference standard for choroidal filling	Invasive; not repeatable at short intervals; no depth resolution, so the CC is not separable from the deeper choroid; largely qualitative
Structural OCT (EDI/swept-source)	Choroidal thickness; large vessel architecture; pachyvessels	Widely available; fast; reproducible thickness measurement	Aggregates lumen and stroma; varies with age, axial length and time of day; does not isolate the CC
Choroidal vascularity index	Ratio of luminal to total choroidal area on binarized B-scans	Less dependent on axial length and diurnal variation than thickness alone	Binarization-dependent; whole-choroid metric; no CC-specific information
OCT angiography (spectral domain)	En face CC flow signal and flow deficits	Non-invasive and repeatable; widely deployed	Limited penetration beneath the RPE; greater susceptibility to shadowing than SS instruments
OCT angiography (swept-source)	En face CC flow deficits with signal compensation; widefield montage	Better penetration; validated compensation and thresholding; quantitative FD metrics	Device- and software-dependent values; artefact-prone under drusen, fluid and atrophy; limited normative data
Adjunctive functional testing	Microperimetry, dark adaptation, low-luminance acuity	Relates flow deficits to spatially matched retinal function	Time-consuming; no accepted protocol for pairing with CC mapping

CC, choriocapillaris; CVI, choroidal vascularity index; EDI, enhanced depth imaging; FD, flow deficit; ICGA, indocyanine green angiography; OCT, optical coherence tomography; OCTA, OCT angiography; RPE, retinal pigment epithelium; SS, swept source. Cross-references to the main text: ICGA, Section 4.1; structural OCT and CVI, Section 4.2; OCTA slab definition, compensation and metrics, Section 4.3 and Section 4.4; artefacts and standardization, Section 4.5, Section 4.6 and Section 4.7; adjunctive functional testing, Section 13.

**Table 2:** Choriocapillaris findings across the principal chorioretinal diseases discussed in this review.

Disease	Choriocapillaris Finding	Proposed Mechanism	Clinical Relevance
Normal aging	Progressive increase in FD percentage and mean FD size, greatest centrally	Capillary dropout, luminal narrowing, Bruch’s membrane thickening	Establishes the baseline; age matching is mandatory in every comparison
Early and intermediate AMD	Increased FD, present beyond the areas occupied by drusen	Complement-mediated endothelial injury; loss of RPE trophic support	Baseline impairment associated with subsequent drusen formation and enlargement (single cohort) and with progression to advanced disease
Subretinal drusenoid deposits	Marked FD, greater than with conventional drusen	High rod metabolic demand; regional CC vulnerability	Correlates with reduced visual acuity; associated with higher progression risk
Geographic atrophy	Severe FD within the lesion; increased FD in the junctional zone	Loss of overlying RPE; primary vascular compromise at the margin	Perilesional FD associated with enlargement rate; candidate stratification variable
Macular neovascularization	Increased FD adjacent to the lesion; subclinical type 1 MNV detectable	Localized ischemia as a stimulus for neovascular growth	Detection before exudation; relationship with FD probably reciprocal
Pachychoroid spectrum and CSC	Focal FD overlying dilated Haller layer vessels	Proposed: mechanical compression by pachyvessels; venous overload (indirect evidence)	Artefact from subretinal fluid is a major confounder; findings extend to fellow eyes
Diabetic retinopathy	Increased FD reported before visible retinopathy (not confirmed in all studies), worsening with severity	Diabetic choroidopathy: capillary dropout and basement membrane thickening	May explain outer retinal damage unexplained by retinal non-perfusion; longitudinal data limited
Pathological myopia	Reduced CC flow signal, increasing with axial length	Choroidal thinning and mechanical stretching	Contributes to atrophic lesions and myopic MNV; requires magnification correction
Inherited retinal disease	FD accompanying and closely matching outer retinal degeneration	Predominantly secondary loss of trophic support; primary in choroideremia	Relevant to eligibility for gene and cell therapy
Inflammatory chorioretinopathies	Well-demarcated FD matching ICGA hypofluorescence; partial reperfusion on resolution	Inflammatory choriocapillary occlusion	Supports a primary choriocapillary process; repeatable monitoring of treatment response

AMD, age-related macular degeneration; CC, choriocapillaris; CSC, central serous chorioretinopathy; FD, flow deficit; MNV, macular neovascularization; RPE, retinal pigment epithelium.

**Table 3:** Methodological requirements for reporting quantitative choriocapillaris measurements, with the consequence of omitting each.

Requirement	Why it Matters	Consequence if Omitted
Explicit slab definition relative to Bruch’s membrane	Anterior slabs include RPE signal and projection artefact; posterior slabs include Sattler layer	Values not comparable with any other study; systematic over- or underestimation of FD
Validated signal compensation	RPE reflectivity varies with pigment, drusen and atrophy, modulating the signal reaching the CC	Spurious deficits beneath drusen; genuine deficits masked over atrophic RPE
Threshold derived from a normal database	Image-specific thresholds are not transferable between eyes	Between-eye and between-study comparison invalid
Exclusion of deficits below normal intercapillary spacing	Small dark regions represent normal anatomy rather than dropout	Physiological intercapillary spacing counted as disease
Reporting of scan size and analyzed region	Transverse sampling density and regional FD both vary with scan size and eccentricity	3 × 3 mm and 6 × 6 mm values wrongly pooled; regional gradients obscured
Registration and averaging where feasible	Improves signal-to-noise and reduces the influence of single-acquisition artefact	Greater measurement variance; reduced sensitivity to longitudinal change
Magnification correction in myopic eyes	A nominal scan subtends a larger retinal area in a longer eye	Systematic bias in any comparison involving differing axial lengths
Quality control and explicit exclusion criteria	Media opacity, low signal strength, motion and segmentation failure degrade the measurement	Poor-quality scans bias FD upwards, disproportionately in advanced disease
Age matching and adjustment for systemic vascular comorbidity	FD rises with age and is influenced by hypertension, diabetes and smoking	Disease effect confounded by aging and systemic vascular status

CC, choriocapillaris; FD, flow deficit; RPE, retinal pigment epithelium. Cross-references to the main text: slab definition, compensation and threshold derivation, Section 4.3; exclusion of small deficits, scan size and averaging, Section 4.4; quality control and artefacts, Section 4.5; magnification correction, Section 9; age matching and systemic adjustment, Section 5 and Section 12.

## Data Availability

No new data were created or analyzed in this study. Data sharing is not applicable to this article.

## Abbreviations

The following abbreviations are used in this manuscript:

AMD	Age-related macular degeneration
BrM	Bruch’s membrane
CC	Choriocapillaris
CSC	Central serous chorioretinopathy
CVI	Choroidal vascularity index
FD	Flow deficit
GA	Geographic atrophy
ICGA	Indocyanine green angiography
MAC	Membrane attack complex
MNV	Macular neovascularization
OCT	Optical coherence tomography
OCTA	Optical coherence tomography angiography
RPE	Retinal pigment epithelium
SDD	Subretinal drusenoid deposits
SS	Swept source
VEGF	Vascular endothelial growth factor

## References

[1] Nickla D.L., Wallman J. (2010). The multifunctional choroid. Prog. Retin. Eye Res..

[2] Torczynski E., Tso M.O. (1976). The architecture of the choriocapillaris at the posterior pole. Am. J. Ophthalmol..

[3] Ramrattan R.S., van der Schaft T.L., Mooy C.M., de Bruijn W.C., Mulder P.G., de Jong P.T. (1994). Morphometric analysis of Bruch’s membrane, the choriocapillaris, and the choroid in aging. Investig. Ophthalmol. Vis. Sci..

[4] Spaide R.F. (2016). Choriocapillaris flow features follow a power law distribution: Implications for characterization and mechanisms of disease progression. Am. J. Ophthalmol..

[5] Chu Z., Zhang Q., Gregori G., Rosenfeld P.J., Wang R.K. (2021). Guidelines for imaging the choriocapillaris using OCT angiography. Am. J. Ophthalmol..

[6] Baethge C., Goldbeck-Wood S., Mertens S. (2019). SANRA—A scale for the quality assessment of narrative review articles. Res. Integr. Peer Rev..

[7] Zhang Q., Zheng F., Motulsky E.H., Gregori G., Chu Z., Chen C.L., Li C., de Sisternes L., Durbin M., Rosenfeld P.J. (2018). A novel strategy for quantifying choriocapillaris flow voids using swept-source OCT angiography. Investig. Ophthalmol. Vis. Sci..

[8] Zhang Q., Shi Y., Zhou H., Gregori G., Chu Z., Zheng F., Motulsky E.H., de Sisternes L., Durbin M., Rosenfeld P.J. (2018). Accurate estimation of choriocapillaris flow deficits beyond normal intercapillary spacing with swept source OCT angiography. Quant. Imaging Med. Surg..

[9] Blaauwgeers H.G., Holtkamp G.M., Rutten H., Witmer A.N., Koolwijk P., Partanen T.A., Alitalo K., Kroon M.E., Kijlstra A., van Hinsbergh V.W. (1999). Polarized vascular endothelial growth factor secretion by human retinal pigment epithelium and localization of vascular endothelial growth factor receptors on the inner choriocapillaris. Am. J. Pathol..

[10] Saint-Geniez M., Kurihara T., Sekiyama E., Maldonado A.E., D’Amore P.A. (2009). An essential role for RPE-derived soluble VEGF in the maintenance of the choriocapillaris. Proc. Natl. Acad. Sci. USA.

[11] Mullins R.F., Schoo D.P., Sohn E.H., Flamme-Wiese M.J., Workamelahu G., Johnston R.M., Wang K., Tucker B.A., Stone E.M. (2014). The membrane attack complex in aging human choriocapillaris: Relationship to macular degeneration and choroidal thinning. Am. J. Pathol..

[12] Whitmore S.S., Sohn E.H., Chirco K.R., Drack A.V., Stone E.M., Tucker B.A., Mullins R.F. (2015). Complement activation and choriocapillaris loss in early AMD: Implications for pathophysiology and therapy. Prog. Retin. Eye Res..

[13] Spraul C.W., Lang G.E., Grossniklaus H.E. (1996). Morphometric analysis of the choroid, Bruch’s membrane, and retinal pigment epithelium in eyes with age-related macular degeneration. Investig. Ophthalmol. Vis. Sci..

[14] Spaide R.F., Gemmy Cheung C.M., Matsumoto H., Kishi S., Boon C.J.F., van Dijk E.H.C., Mauget-Faysse M., Behar-Cohen F., Hartnett M.E., Sivaprasad S. (2022). Venous overload choroidopathy: A hypothetical framework for central serous chorioretinopathy and allied disorders. Prog. Retin. Eye Res..

[15] Biesemeier A., Taubitz T., Julien S., Yoeruek E., Schraermeyer U. (2014). Choriocapillaris breakdown precedes retinal degeneration in age-related macular degeneration. Neurobiol. Aging.

[16] Mullins R.F., Johnson M.N., Faidley E.A., Skeie J.M., Huang J. (2011). Choriocapillaris vascular dropout related to density of drusen in human eyes with early age-related macular degeneration. Investig. Ophthalmol. Vis. Sci..

[17] Spaide R.F., Fujimoto J.G., Waheed N.K., Sadda S.R., Staurenghi G. (2018). Optical coherence tomography angiography. Prog. Retin. Eye Res..

[18] Borrelli E., Sarraf D., Freund K.B., Sadda S.R. (2018). OCT angiography and evaluation of the choroid and choroidal vascular disorders. Prog. Retin. Eye Res..

[19] Agrawal R., Gupta P., Tan K.A., Cheung C.M., Wong T.Y., Cheng C.Y. (2016). Choroidal vascularity index as a measure of vascular status of the choroid: Measurements in healthy eyes from a population-based study. Sci. Rep..

[20] Byon I., Nassisi M., Borrelli E., Sadda S.R. (2019). Impact of slab selection on the quantification of choriocapillaris flow deficits by optical coherence tomography angiography. Am. J. Ophthalmol..

[21] Mehta N., Liu K., Alibhai A.Y., Gendelman I., Braun P.X., Ishibazawa A., Sorour O., Duker J.S., Waheed N.K. (2019). Impact of binarization thresholding and brightness/contrast adjustment methodology on optical coherence tomography angiography image quantification. Am. J. Ophthalmol..

[22] Uji A., Balasubramanian S., Lei J., Baghdasaryan E., Al-Sheikh M., Sadda S.R. (2017). Choriocapillaris imaging using multiple en face optical coherence tomography angiography image averaging. JAMA Ophthalmol..

[23] Chu Z., Zhou H., Cheng Y., Zhang Q., Wang R.K. (2018). Improving visualization and quantitative assessment of choriocapillaris with swept source OCTA through registration and averaging applicable to clinical systems. Sci. Rep..

[24] Chu Z., Gregori G., Rosenfeld P.J., Wang R.K. (2019). Quantification of choriocapillaris with optical coherence tomography angiography: A comparison study. Am. J. Ophthalmol..

[25] Zheng F., Zhang Q., Shi Y., Russell J.F., Motulsky E.H., Banta J.T., Chu Z., Zhou H., Patel N.A., de Sisternes L. (2019). Age-dependent changes in the macular choriocapillaris of normal eyes imaged with swept-source optical coherence tomography angiography. Am. J. Ophthalmol..

[26] Borrelli E., Uji A., Toto L., Viggiano P., Evangelista F., Mastropasqua R. (2019). In vivo mapping of the choriocapillaris in healthy eyes: A widefield swept-source OCT angiography study. Ophthalmol. Retin..

[27] Borrelli E., Shi Y., Uji A., Balasubramanian S., Nassisi M., Sarraf D., Sadda S.R. (2018). Topographic analysis of the choriocapillaris in intermediate age-related macular degeneration. Am. J. Ophthalmol..

[28] Nassisi M., Tepelus T., Nittala M.G., Sadda S.R. (2019). Choriocapillaris flow impairment predicts the development and enlargement of drusen. Graefes Arch. Clin. Exp. Ophthalmol..

[29] Corvi F., Tiosano L., Corradetti G., Nittala M.G., Lindenberg S., Alagorie A.R., McLaughlin J.A., Lee T.K., Sadda S.R. (2021). Choriocapillaris flow deficits as a risk factor for progression of age-related macular degeneration. Retina.

[30] Tiosano L., Corradetti G., Sadda S.R. (2021). Progression of choriocapillaris flow deficits in clinically stable intermediate age-related macular degeneration. Eye.

[31] Johari Moghadam M.M., Montazeri F., Feldman S., Lee S.C., Yiu G., Moshiri A., Emami-Naeini P., Moussa K., Park S.S. (2025). Longitudinal choriocapillaris and retinal vascular flow changes on OCTA with progression to advanced AMD. Clin. Ophthalmol..

[32] Romano F., Ding X., Yuan M., Vingopoulos F., Garg I., Choi H., Alvarez R., Tracy J.H., Finn M., Razavi P. (2024). Progressive Choriocapillaris Changes on Optical Coherence Tomography Angiography Correlate With Stage Progression in AMD. Investig. Ophthalmol. Vis. Sci..

[33] Nesper P.L., Soetikno B.T., Fawzi A.A. (2017). Choriocapillaris nonperfusion is associated with poor visual acuity in eyes with reticular pseudodrusen. Am. J. Ophthalmol..

[34] Alten F., Heiduschka P., Clemens C.R., Eter N. (2016). Exploring choriocapillaris under reticular pseudodrusen using OCT-angiography. Graefes Arch. Clin. Exp. Ophthalmol..

[35] Thulliez M., Zhang Q., Shi Y., Zhou H., Chu Z., de Sisternes L., Durbin M.K., Feuer W., Gregori G., Wang R.K. (2019). Correlations between choriocapillaris flow deficits around geographic atrophy and enlargement rates based on swept-source OCT imaging. Ophthalmol. Retin..

[36] Nassisi M., Baghdasaryan E., Borrelli E., Ip M., Sadda S.R. (2019). Choriocapillaris flow impairment surrounding geographic atrophy correlates with disease progression. PLoS ONE.

[37] Alagorie A.R., Nassisi M., Verma A., Nittala M., Corradetti G., Velaga S., Sadda S.R. (2020). Relationship between proximity of choriocapillaris flow deficits and enlargement rate of geographic atrophy. Graefes Arch. Clin. Exp. Ophthalmol..

[38] Moult E.M., Shi Y., Zhang Q., Wang L., Mazumder R., Chen S., Chu Z., Feuer W., Waheed N.K., Gregori G. (2021). Analysis of correlations between local geographic atrophy growth rates and local OCT angiography-measured choriocapillaris flow deficits. Biomed. Opt. Express.

[39] Sacconi R., Corbelli E., Carnevali A., Mercuri S., Rabiolo A., Marchese A., Cicinelli M.V., Bandello F., Querques G. (2021). Choriocapillaris flow impairment could predict the enlargement of geographic atrophy lesion. Br. J. Ophthalmol..

[40] Spaide R.F., Jaffe G.J., Sarraf D., Freund K.B., Sadda S.R., Staurenghi G., Waheed N.K., Chakravarthy U., Rosenfeld P.J., Holz F.G. (2020). Consensus nomenclature for reporting neovascular age-related macular degeneration data: Consensus on Neovascular Age-Related Macular Degeneration Nomenclature Study Group. Ophthalmology.

[41] Treister A.D., Nesper P.L., Fayed A.E., Gill M.K., Mirza R.G., Fawzi A.A. (2018). Prevalence of subclinical CNV and choriocapillaris nonperfusion in fellow eyes of unilateral exudative AMD on OCT angiography. Transl. Vis. Sci. Technol..

[42] Toma C., Gatti V., Servillo A., Galotta A., Leonardi M., Ferrante D., Torti E., Leporati F., De Cillà S. (2025). Early choriocapillaris dysfunction in fellow eyes of patients with unilateral neovascular age-related macular degeneration. Eur. J. Ophthalmol..

[43] Dansingani K.K., Balaratnasingam C., Naysan J., Freund K.B. (2016). En face imaging of pachychoroid spectrum disorders with swept-source optical coherence tomography. Retina.

[44] Cheung C.M.G., Lee W.K., Koizumi H., Dansingani K., Lai T.Y.Y., Freund K.B. (2019). Pachychoroid disease. Eye.

[45] Rochepeau C., Kodjikian L., Garcia M.A., Coulon C., Burillon C., Denis P., Delaunay B., Mathis T. (2018). Optical coherence tomography angiography quantitative assessment of choriocapillaris blood flow in central serous chorioretinopathy. Am. J. Ophthalmol..

[46] Baek J., Kook L., Lee W.K. (2019). Choriocapillaris flow impairments associated with pachyvessel in early pachychoroid. Sci. Rep..

[47] Meng Y., Xu Y., Li L., Su Y., Zhang L., Chen C., Yi Z. (2022). Wide-field OCT-angiography assessment of choroidal thickness and choriocapillaris in eyes with central serous chorioretinopathy. Front. Physiol..

[48] Nesper P.L., Roberts P.K., Onishi A.C., Chai H., Liu L., Jampol L.M., Fawzi A.A. (2017). Quantifying microvascular abnormalities with increasing severity of diabetic retinopathy using optical coherence tomography angiography. Investig. Ophthalmol. Vis. Sci..

[49] Choi W., Waheed N.K., Moult E.M., Adhi M., Lee B., De Carlo T., Jayaraman V., Baumal C.R., Duker J.S., Fujimoto J.G. (2017). Ultrahigh speed swept source optical coherence tomography angiography of retinal and choriocapillaris alterations in diabetic patients with and without retinopathy. Retina.

[50] Wang J., Hormel T., Park D.W., Haq S., Bailey S., Huang D., Hwang T.S., Jia Y. (2025). Quantifying choriocapillaris flow deficits in diabetic retinopathy using projection-resolved OCT angiography. Investig. Ophthalmol. Vis. Sci..

[51] Dodo Y., Suzuma K., Ishihara K., Yoshitake S., Fujimoto M., Yoshitake T., Miwa Y., Murakami T. (2017). Clinical relevance of reduced decorrelation signals in the diabetic inner choroid on optical coherence tomography angiography. Sci. Rep..

[52] Nagai N., Mushiga Y., Ozawa Y. (2023). Diabetic choriocapillaris flow deficits affect the outer retina and are related to hemoglobin A1c and systolic blood pressure levels. Sci. Rep..

[53] Al-Sheikh M., Phasukkijwatana N., Dolz-Marco R., Rahimi M., Iafe N.A., Freund K.B., Sadda S.R., Sarraf D. (2017). Quantitative OCT angiography of the retinal microvasculature and the choriocapillaris in myopic eyes. Investig. Ophthalmol. Vis. Sci..

[54] Ohno-Matsui K., Kawasaki R., Jonas J.B., Cheung C.M., Saw S.M., Verhoeven V.J., Klaver C.C., Moriyama M., Shinohara K., Kawasaki Y. (2015). International photographic classification and grading system for myopic maculopathy. Am. J. Ophthalmol..

[55] Battaglia Parodi M., Cicinelli M.V., Rabiolo A., Pierro L., Gagliardi M., Bolognesi G., Bandello F. (2017). Vessel density analysis in patients with retinitis pigmentosa by means of optical coherence tomography angiography. Br. J. Ophthalmol..

[56] Mastropasqua R., Toto L., Borrelli E., Di Antonio L., Mattei P.A., Senatore A., Di Nicola M., Mariotti C. (2017). Optical coherence tomography angiography findings in Stargardt disease. PLoS ONE.

[57] Jain N., Jia Y., Gao S.S., Zhang X., Weleber R.G., Huang D., Pennesi M.E. (2016). Optical coherence tomography angiography in choroideremia: Correlating choriocapillaris loss with overlying degeneration. JAMA Ophthalmol..

[58] Klufas M.A., Phasukkijwatana N., Iafe N.A., Prasad P.S., Agarwal A., Gupta V., Ansari W., Pichi F., Souza E., Neri P. (2017). Optical coherence tomography angiography reveals choriocapillaris flow reduction in placoid chorioretinitis. Ophthalmol. Retin..

[59] Burke T.R., Chu C.J., Salvatore S., Bailey C., Dick A.D., Lee R.W.J., Ross A.H., Carreno E. (2017). Application of OCT-angiography to characterise the evolution of chorioretinal lesions in acute posterior multifocal placoid pigment epitheliopathy. Eye.

[60] Zahid S., Chen K.C., Jung J.J., Balaratnasingam C., Ghadiali Q., Sorenson J., Rofagha S., Freund K.B., Yannuzzi L.A. (2017). Optical coherence tomography angiography of chorioretinal lesions due to idiopathic multifocal choroiditis. Retina.

[61] Aggarwal K., Agarwal A., Mahajan S., Invernizzi A., Mandadi S.K.R., Singh R., Bansal R., Dogra M.R., Gupta V., OCTA Study Group (2018). The role of optical coherence tomography angiography in the diagnosis and management of acute Vogt-Koyanagi-Harada disease. Ocul. Immunol. Inflamm..

[62] Chu Z., Weinstein J.E., Wang R.K., Pepple K.L. (2020). Quantitative analysis of the choriocapillaris in uveitis using en face swept-source optical coherence tomography angiography. Am. J. Ophthalmol..

[63] Spaide R.F. (2017). Choriocapillaris signal voids in maternally inherited diabetes and deafness and in pseudoxanthoma elasticum. Retina.

[64] Chua J., Chin C.W.L., Hong J., Chee M.L., Le T.T., Ting D.S.W., Wong T.Y., Schmetterer L. (2019). Impact of hypertension on retinal capillary microvasculature using optical coherence tomographic angiography. J. Hypertens..

[65] Wagner S.K., Fu D.J., Faes L., Liu X., Huemer J., Khalid H., Ferraz D., Korot E., Kelly C., Balaskas K. (2020). Insights into systemic disease through retinal imaging-based oculomics. Transl. Vis. Sci. Technol..

[66] Han I.C., Tadarati M., Pacheco K.D., Scott A.W. (2017). Evaluation of macular vascular abnormalities identified by optical coherence tomography angiography in sickle cell disease. Am. J. Ophthalmol..

[67] Mihailovic N., Lahme L., Braasch S., Rosenberger F., Eter N., Ehrchen J., Alnawaiseh M. (2022). Altered ocular microvasculature in patients with systemic sclerosis and very early disease of systemic sclerosis using optical coherence tomography angiography. Sci. Rep..

[68] Kılınç Hekimsoy H., Şekeroğlu M.A., Koçer A.M., Akdoğan A. (2020). Analysis of retinal and choroidal microvasculature in systemic sclerosis: An optical coherence tomography angiography study. Eye.

[69] Pfau M., von der Emde L., de Sisternes L., Hallak J.A., Leng T., Schmitz-Valckenberg S., Holz F.G., Fleckenstein M., Rubin D.L. (2020). Progression of photoreceptor degeneration in geographic atrophy secondary to age-related macular degeneration. JAMA Ophthalmol..

[70] Nassisi M., Tepelus T., Corradetti G., Sadda S.R. (2021). Relationship between choriocapillaris flow and scotopic microperimetry in early and intermediate age-related macular degeneration. Am. J. Ophthalmol..

[71] Corradetti G., Tiosano L., Nassisi M., Alagorie A.R., Corvi F., Nittala M.G., Sadda S. (2021). Scotopic microperimetric sensitivity and inner choroid flow deficits as predictors of progression to nascent geographic atrophy. Br. J. Ophthalmol..

[72] Kar D., Corradetti G., Swain T.A., Clark M.E., McGwin G., Owsley C., Sadda S.R., Curcio C.A. (2023). Choriocapillaris Impairment Is Associated With Delayed Rod-Mediated Dark Adaptation in Age-Related Macular Degeneration. Investig. Ophthalmol. Vis. Sci..

[73] Rinella N.T., Zhou H., Wong J., Zhang Q., Nattagh K., Porco T.C., Wang R.K., Schwartz D.M., Duncan J.L. (2022). Correlation between localized choriocapillaris perfusion and macular function in eyes with geographic atrophy. Am. J. Ophthalmol..

[74] Sadda S.R., Guymer R., Holz F.G., Schmitz-Valckenberg S., Curcio C.A., Bird A.C., Blodi B.A., Bottoni F., Chakravarthy U., Chew E.Y. (2018). Consensus definition for atrophy associated with age-related macular degeneration on OCT: Classification of Atrophy Report 3. Ophthalmology.

[75] Guymer R.H., Rosenfeld P.J., Curcio C.A., Holz F.G., Staurenghi G., Freund K.B., Schmitz-Valckenberg S., Sparrow J., Spaide R.F., Tufail A. (2020). Incomplete retinal pigment epithelial and outer retinal atrophy in age-related macular degeneration: Classification of Atrophy Meeting Report 4. Ophthalmology.

[76] Liao D.S., Grossi F.V., El Mehdi D., Gerber M.R., Brown D.M., Heier J.S., Wykoff C.C., Singerman L.J., Abraham P., Grassmann F. (2020). Complement C3 inhibitor pegcetacoplan for geographic atrophy secondary to age-related macular degeneration: A randomized phase 2 trial. Ophthalmology.

[77] Heier J.S., Lad E.M., Holz F.G., Rosenfeld P.J., Guymer R.H., Boyer D., Grossi F., Baumal C.R., Korobelnik J.F., Slakter J.S. (2023). Pegcetacoplan for the treatment of geographic atrophy secondary to age-related macular degeneration (OAKS and DERBY): Two multicentre, randomised, double-masked, sham-controlled, phase 3 trials. Lancet.

[78] Khanani A.M., Patel S.S., Staurenghi G., Tadayoni R., Danzig C.J., Eichenbaum D.A., Hsu J., Wykoff C.C., Heier J.S., Lally D.R. (2023). Efficacy and safety of avacincaptad pegol in patients with geographic atrophy (GATHER2): 12-month results from a randomised, double-masked, phase 3 trial. Lancet.

[79] Pfau M., Schmitz-Valckenberg S., Ribeiro R., Safaei R., McKeown A., Fleckenstein M., Holz F.G. (2022). Association of complement C3 inhibitor pegcetacoplan with reduced photoreceptor degeneration beyond areas of geographic atrophy. Sci. Rep..

[80] van Dijk E.H.C., Fauser S., Breukink M.B., Blanco-Garavito R., Groenewoud J.M.M., Keunen J.E.E., Peters P.J.H., Dijkman G., Souied E.H., MacLaren R.E. (2018). Half-dose photodynamic therapy versus high-density subthreshold micropulse laser treatment in patients with chronic central serous chorioretinopathy: The PLACE trial. Ophthalmology.

[81] Hikichi T., Agarie M. (2019). Reduced vessel density of the choriocapillaris during anti-vascular endothelial growth factor therapy for neovascular age-related macular degeneration. Investig. Ophthalmol. Vis. Sci..

[82] Viggiano P., Grassi M.O., Pignataro M., Boscia G., Borrelli E., Molfetta T., Evangelista F., Alessio G., Boscia F. (2022). Topographical analysis of the choriocapillaris reperfusion after loading anti-VEGF therapy in neovascular AMD. Transl. Vis. Sci. Technol..

[83] Brinkmann M., Viggiano P., Boscia G., Müller T., Castellino N., Schweighofer J., Boscia F., Toro M.D., El-Shabrawi Y. (2024). Analysis of choriocapillaris reperfusion topography following faricimab treatment for neovascular age-related macular degeneration in therapy-naïve patients. Ophthalmol. Ther..

[84] Schmidt-Erfurth U., Sadeghipour A., Gerendas B.S., Waldstein S.M., Bogunović H. (2018). Artificial intelligence in retina. Prog. Retin. Eye Res..

[85] Zhou Y., Chia M.A., Wagner S.K., Ayhan M.S., Williamson D.J., Struyven R.R., Liu T., Xu M., Lozano M.G., Woodward-Court P. (2023). A foundation model for generalizable disease detection from retinal images. Nature.

